# Analysis of Metastases and Second Primary Malignancy Development in Patients with Invasive Transitional Cell Carcinoma of the Bladder

**DOI:** 10.3390/cancers17162663

**Published:** 2025-08-15

**Authors:** Keren Rouvinov, Alexander Yakobson, Angela Tiganas, Noa Shani Shrem, Elena Chernomordikov, Ashraf Abu Jama, Nashat Abu Yasin, Ronen Brenner, Anna Ievko, Ez El Din Abu Zeid, Mhammad Abu Juda, Walid Shalata

**Affiliations:** 1 The Legacy Heritage Cancer Center, Dr. Larry Norton Institute, Soroka Medical Center, Beer-Sheva 84105, Israelnoashs@clalit.org.il (N.S.S.);; 2 Faculty of Health Sciences, Ben-Gurion University of the Negev, Beer Sheva 84105, Israelchernomordikova@gmail.com (E.C.); abuzeid@post.bgu.ac.il (E.E.D.A.Z.);; 3 Edith Wolfson Medical Center, Oncology Institute, Holon 58220, Israel; ronen.brenner@gmail.com (R.B.);

**Keywords:** transitional cell carcinoma, bladder, metastases, second primary malignancies, cancer

## Abstract

**Background:** Invasive BC patients are at risk of loco-regional recurrence, distant MTS, and the development of second primary tumors. SPMs comprise the sixth most common group of malignancies. **Material and methods:** The records of 125 consecutive patients with primary invasive TCC of the bladder seen in the Oncology Department of Soroka University Medical Center were reviewed between January 2016 and December 2023. We recorded demographic details, the type of primary treatment, tumor site, time to diagnosis of MTS, and occurrence of SPMs. **Results:** The primary treatments included RC in 58 patients (median age 66 years, range 43–86), PC in 9 patients (median age 64 years, range 22–73), and XRT in 23 patients (median age 74 years, range 22–87). Five patients from the PC group were also treated by XRT. A total of 90 (72%) patients developed MTS or SPMs, with 66 of these developing MTS and 24 developing SPMs. The median age was 70 years (range 22–87). The most frequent site of MTS was in the pelvic LNs (34 patients), followed by bone (18 patients), liver (8 patients), and lung (6 patients), with 4 patients developing synchronous MTS in the pelvic LNs and liver. The median time from diagnosis to MTS was 14.3 months. The distribution of MTS varied according to primary treatment. After RC, 17 patients developed LN MTS, 7 liver, 6 bone, and 3 lung MTS. The average times for developing MTS were as follows: LNs, 14.8 months, liver, 59.7 months, bone, 6.8 months, and lung, 16 months. Following XRT, LN MTS developed in 17 patients: 12 bone, 3 lung, and 1 liver. The most frequent SPMs were prostate cancer with 11 patients and lung cancer with 6 patients, with the median time from TCC diagnosis of 54 months. **Conclusion:** A regular extended follow-up for invasive BC patients is vital to ensure the early detection of frequently occurring MTS and SPMs. Through the early diagnosis of local recurrences, MTS, and SPMs, treatment results and patient prognosis can be significantly improved.

## Simple Summary

Invasive bladder cancer (BC) poses a risk for local recurrence, distant metastases (MTS), and second primary malignancies (SPMs), which are among the most common cancer-related complications. This study reviewed the records of 125 patients treated for invasive transitional cell carcinoma (TCC) of the bladder at the Soroka University Medical Center between 2016 and 2023. Patients received radical cystectomy (RC), partial cystectomy (PC), or radiotherapy (XRT). Over 70% of the patients developed either MTS or SPMs. The pelvic lymph nodes (LNs) were the most common site of MTS, followed by bone, liver, and lung. The average time to metastasis varied by organ and treatment type. SPMs most frequently involved the prostate and lungs. These findings highlight the importance of long-term follow-up for patients with invasive BC. Timely monitoring may allow earlier detection of disease progression or new cancers, potentially improving treatment outcomes and survival rates. Regular surveillance should be an essential component of post-treatment care.

## 1. Introduction

Cancer is a significant public health issue, accounting for nearly one in six deaths (16.8%) overall and one in four deaths (22.8%) with regard to non-communicable diseases (NCDs) globally [1,2,3,4]. The disease is responsible for 30.3% of premature NCD-related deaths, affecting those aged 30 to 69 years, and ranks among the top three causes of death in this age group in most countries [1,2,3,4,5,6,7].

TCC, previously known as urothelial carcinoma (UC), is the fourth most common cancer globally [2,3,4,5,6,7]. In the United States, it ranks as the fourth most common cancer among men and the tenth most common among women. Studies have shown a higher incidence of BC in males versus females, with the ratio ranging from 2:1 to 3:1 [4,5,6,7,8,9]. The American Cancer Society estimates that there will be approximately 81,190 new cases of BC and 17,240 related deaths [4,5,6,7,8,9,10,11,12,13].

TCC is a malignant cancer that originates in the transitional epithelial cells of the urinary tract, accounting for approximately 95% of all BCs [5]. The remaining 5% consists of squamous cell carcinoma, adenocarcinoma, and small cell carcinoma [1,3,4,5,14,15]. TCC is also the most common cancer type found in the urinary tract, occurring in both the lower (bladder and urethra) and the upper (renal pelvis and ureter) parts of the tract. TCC is more common in older adults, with the median age of diagnosis being 72 years for men and 75 years for women [14,15]. Additionally, TCC shows significant racial disparities where its incidence is twice as high in Caucasians compared to African Americans, for yet to be known reasons [14,15,16].

Several environmental factors have been linked to the development of TCC. The most well-established risk factor is cigarette smoking, which is responsible for approximately 55% of all cases in the United States [17,18,19,20]. In smokers, aromatic amines are the primary carcinogens associated with BC [18,19,20,21,22,23]. Following smoking, occupational exposure to various carcinogens, such as polycyclic aromatic hydrocarbons and chlorinated hydrocarbons, accounts for approximately 20% of cases, particularly in industrial areas where paint and dye are processed [19,20,21,22]. Obesity has also been identified as a major risk factor for TCC [17,18,19,20,21,22,23,24]. A meta-analysis of 15 cohort studies, which included over 38,000 TCC patients, found a 4.2% increase in the incidence of BC for every 5 kg/m^2^ increase in body mass index (BMI) among patients [24].

Major risk factors persist after the primary treatment of invasive cancer, including locoregional recurrence, distant MTS, and the development of SPMs [25,26]. SPMs are the sixth most common type of cancer among cancer patients. Additionally, about 10% of all new cancer diagnoses occur in cancer survivors, with 8% of this population having suffered from cancer more than once [25,26]. Unfortunately, there are no specific or reliable methods for the early detection of recurrence or metastasis of BC. Marker screening and diagnostic tools do not always provide reliable information for the early detection of recurrence or metastasis of BC, necessitating the use of specialized techniques—such as imaging, blood tests, and genetic testing—to improve surveillance accuracy and guide timely clinical intervention. And these diagnostic tools are usually employed when the patient shows symptoms. Even when the initial cancer is under control, the development of SPMs can result in poor survival outcomes [25,26].

Although most cancer patients are regularly monitored, only a small percentage of recurrences, MTS, and second primary cancers are detected during these follow-ups. In most cases, these diagnoses are made only when patients become symptomatic or as incidental findings during tests unrelated to the original cancer [27,28].

This study examined the patterns of local recurrence, MTS, and SPMs in a cohort of patients with invasive TCC of the bladder. The goal was to inform and optimize follow-up strategies and enhance early diagnosis, ultimately improving treatment outcomes.

## 2. Patients and Methods

### 2.1. Study Population

In this retrospective study, the records of 125 consecutive patients diagnosed with primary invasive TCC of the bladder and treated at the Soroka University Medical Center between January 2016 and December 2023 were reviewed; 90 patients were eligible for the study, with 72 (80%) of these patients being male and 18 (20%) being female.

### 2.2. Study Design

Demographic information, treatment modalities for the primary tumor, and the site and timing of MTS and SPMs diagnoses were recorded and analyzed. The study also examined patient characteristics, the distribution of MTS, the relationship between MTS and treatment approaches, and the average time for the development of metastasis.

### 2.3. Exclusion Criteria

**Figure 1:**
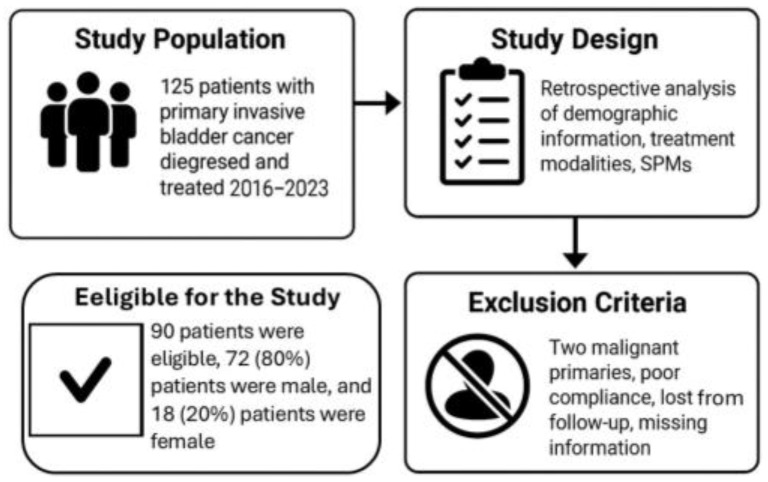
The conclusion of the study population.

Exclusion criteria included patients diagnosed with two malignant primaries: those with poor compliance or lost to follow-up, and patients lacking essential information such as radiological and oncological follow-up data. These criteria were applied to ensure the homogeneity and integrity of the study population, allowing for clearer analysis and interpretation of the research findings (Figure 1).

### 2.4. Statistical Analysis

Descriptive statistics were used to summarize the baseline demographic and clinical characteristics of patients. Continuous data with non-normal distributions were presented as medians (range) while categorical variables were reported as frequencies (percentages). Further analyses were stratified by key clinical characteristics, including primary treatments, sites of MTS, and the distribution of MTS in relation to treatment. Log-rank tests were performed to assess the statistical significance of differences in survival distributions. Multivariable analyses of progression-free survival (PFS) and overall survival (OS) were conducted using Cox proportional hazards regression models to estimate hazard ratios (HRs) along with their corresponding 95% confidence intervals (CIs).

## 3. Results

**Figure 2:**
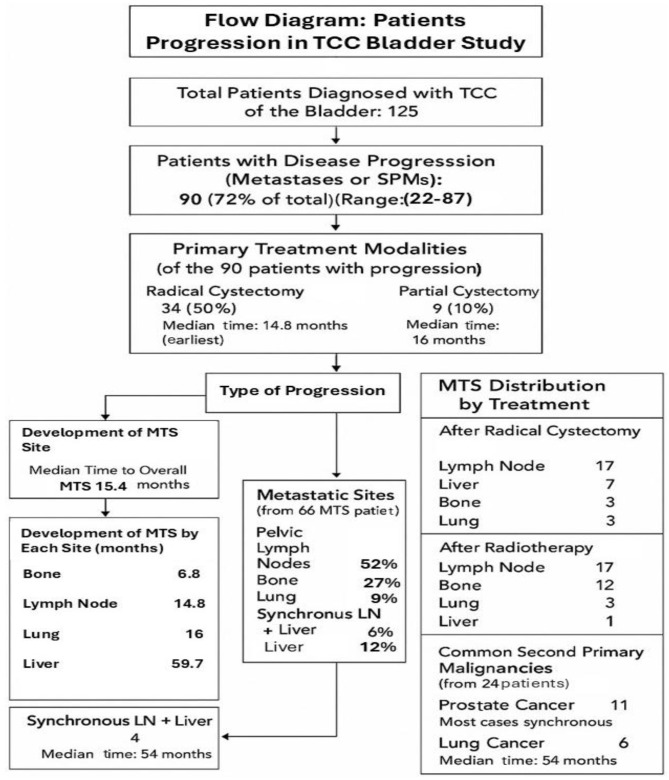
Flow diagram illustrating disease progression, treatment modalities, and metastatic patterns in patients with TCC of the bladder.

Of the 125 patients diagnosed with TCC of the bladder, 90 (72%) developed MTS or SPMs (Figure 2). The characteristics of these 90 patients are shown in Table 1. The median age was 70 years (range 22–87), and the primary treatments included cystectomy in 58 patients (median age 66 years, range 43–86), PC in 9 patients (median age 64 years, range 22–73), and XRT in 23 patients (median age 74 years, range 22–87). Five patients in the PC group also received XRT.

In total, 66 patients developed MTS and 24 developed SPMs. The most common site of metastasis was the pelvic LNs, affecting 34 patients, followed by bone in 18 patients, liver in 8, and lung in 6. Four patients developed synchronous LN and liver MTS. The median time from diagnosis to the development of metastasis was 14.3 months (Table 2).

The distribution of MTS according to treatment is shown in Table 3. After RC, 17 patients developed LN MTS, 7 developed liver MTS, 6 developed bone MTS, and 3 developed lung MTS. Following XRT, 17 patients developed LN MTS, 12 developed bone MTS, 3 developed lung MTS, and 1 developed liver MTS.

The median time to the development of MTS was 6.8 months for bone MTS, 14.8 months for LN MTS, 16 months for lung MTS, and 59.7 months for liver MTS (Table 4) (liver MTS was used as the reference group because it had the longest average time to progression (59.7 months)). The most common SPMs were prostate cancer, diagnosed in 11 patients (with most cases being synchronous), and lung cancer, diagnosed in 6 patients (with a median time of 54 months).

The distribution of metastatic sites between treatment groups revealed no statistically significant differences for LN or lung MTS (*p* = 1.0), while bone MTS showed a non-significant trend toward higher frequency in the XRT group (*p* = 0.10) and liver MTS was more commonly observed after RC, with borderline statistical significance (*p* = 0.05) (Table 5).

## 4. Discussion

TCC, the dominant cancer type of the urinary system, may occur at various sites throughout the urinary tract [1,2,3,4]. While individual malignancies have been extensively studied, research on urethral TCC remains limited, with most studies focusing on case reports. The incidence of TCC in the bladder and urethra has steadily decreased over time, with no significant changes observed in the renal pelvis and ureter [5,6,7,8,9,10,11,12]. The mortality rates for TCC at all four tract sites, however, have shown an increasing trend over the same time period. Changes in smoking rates in the United States may provide an important explanation for this trend. Tobacco smoking is the primary risk factor for TCC, and studies have shown that former smokers have a 2.5 times higher risk of developing malignant TCC compared to non-smokers [15,16,17,18,19,20]. Nearly 55% of new TCC cases in the United States are linked to smoking [17]. According to national health surveys, around 42% of American adults were smokers in 1965, and this rate remained relatively high during the 1970s and 1980s [17,18]. This high smoking prevalence may have contributed to the rising mortality rates. While data from the surveillance, epidemiology, and end results (SEER) database indicates a significant increase in bladder TCC incidence between 1997 and 2007 [4,5,6,28,29], a marked decline in smoking rates among men in North America over recent years may explain the more stable incidence and mortality rates observed toward the end of the study period [8].

Local recurrence, MTS, and SPMs are the three primary causes of death in patients with invasive TCC [8,30,31,32]. Currently, there is no effective treatment to prevent the progression of this disease. While the development of new, more effective drugs continues to be a major research focus, most clinical trials are aimed at treating advanced stages of the disease [31,32]. The successful treatment of recurrent disease or SPMs largely depends on a timely diagnosis, allowing for the earliest initiation of the optimal intervention [31,32].

Invasive TCC of the bladder, particularly muscle-invasive bladder cancer (MIBC), which invades the muscularis propria and beyond, significantly increases the risk of distant metastasis [32,33]. The most common metastatic sites for MIBC include the LNs, bone, the lungs, the liver, and the peritoneum. A previous retrospective study examining patients diagnosed with SPMs between 2000 and 2013 highlighted the strong association between BC and various SPMs [33]. Among BC patients, lung and bronchus cancer was the most frequent SPMs, affecting 9.58% of cases. Prostate cancer was the second most common at 8.83%, followed by colorectal cancer at 5.37%. Other SPMs observed in patients with BC included stomach cancer (6.66%), liver cancer (5.2%), esophageal cancer (6.25%), cervix uteri cancer (5.6%), and thyroid cancer (1.81%). Additionally, non-Hodgkin lymphoma was seen in 4.77% of cases, while pancreatic cancer affected 4.18% and kidney cancer was noted in 10.94%. Corpus uteri cancer accounted for 3.29%, brain and central nervous system cancers were seen in 4.38%, and ovarian cancer in 2.57% [32,33,34,35]. In contrast, our study focused on the metastatic spread of BC and found pelvic LNs to be the most frequent site of MTS (52%), followed by bone (27%), liver (12%), and lung (9%), with 6% of patients having synchronous liver and LN MTS. Notably, while the lung and liver appeared as both common SPMs and metastatic sites, bone MTS were frequent in our study yet not reported among SPMs, highlighting a key distinction between metastatic progression and the development of new primary cancers. Additionally, our study revealed an average time of 14.3 months from initial diagnosis to the development of MTS, underscoring a more aggressive disease course, whereas the previous studies reflected long-term risks of developing second cancers potentially related to shared risk factors such as smoking [32,33,34,35,36]. Overall, while both studies show an overlap in affected organs, they emphasize different aspects of disease evolution [32,36]. The differences between our study and the previous retrospective study on SPMs in BC patients may be attributed to several factors [33,34,35], including geographic, genetic, and lifestyle-related influences. Geographic variation can affect cancer patterns due to differences in environmental exposure, healthcare access, and diagnostic practices, while genetic predispositions may influence tumor aggressiveness and susceptibility to metastasis or multiple primary cancers. Lifestyle behaviors such as smoking, alcohol use, diet, and physical activity also play a significant role as these factors are strongly associated with both BC and many of the SPMs observed in the previous studies such as lung, liver, and colorectal cancers [30,31,32,33,34,35,36]. The relatively short average time to metastasis in our cohort (14.3 months) suggests a more aggressive disease biology or possibly later-stage presentation compared to the earlier study’s population that may have survived longer and thus had more time to develop SPMs. Additionally, differences in surveillance intensity and follow-up protocols between populations could have impacted the detection of second malignancies. Overall, the variations are likely due to a combination of regional, biological, behavioral, and healthcare-related factors.

The European Society of Clinical Oncology (ESMO) and the European Urology Association (EUA) have published guidelines for the follow-up of patients with BC as follows (Table 6):

As a result of the risk of recurrence and progression, patients with non-muscle-invasive bladder carcinoma (NMIBC) need to be followed-up; however, the frequency and duration of cystoscopy and imaging should reflect the individual patient’s degree of risk.

Follow-up and long-term implications are understood according to the ESMO practice guidelines [38,39]. There is no generally accepted follow-up protocol; therefore, the possible options could be as follows: in NMIBC, regular cystoscopy and cytology is mandatory every 3–6 months based on the high or low risk during the first 2 years, and every 6–12 months thereafter to assess tumor response, progression or recurrence.

After definitive treatment of muscle-invasive bladder carcinoma (MIBC) with RC, urine cytology, as well as liver and renal function tests, should be carried out every 3–6 months for 2 years and subsequent to this as clinically indicated. Imaging of the chest, upper tract, abdomen, and pelvis every 3–6 months for 2 years should also be undertaken based on the risk of recurrence and subsequent to this as clinically indicated. Additionally, urethral wash cytology may be carried out every 6–12 months if urethrectomy has not been carried out or if there is a prior history of CIS.

For MIBC patients in whom a bladder preservation strategy has been adopted, there is a need to evaluate the response to treatment after the induction of chemo-radiation. After completion, the same follow-up regimen as for RC is recommended; however, cystoscopy and urine cytology plus random biopsies every 3–6 months for 2 years are necessary. During follow-up, monitoring of long-term treatment toxicities and potential recurrences of secondary tumors should be carried out.

For those who undergo systemic chemotherapy, response evaluation every two to three cycles using the initial radiographic tests carried out during the work-up is also necessary. Providing optimal care for patients also involves addressing psychosocial implications of all the above-mentioned treatment strategies.

As per the above, based on differences in bladder carcinoma specifics, different societies provide different treatment recommendations. The EUA addresses only non-invasive TCC, while ESMO guidelines cover both invasive TCC as well as follow-ups after cystectomy. Both urologists and oncologists should be involved in TCC patient follow-ups, with urologists focusing more on non-invasive disease patients. Cooperation between these two specialties is required for optimal follow-ups. We believe that the time has come for ESMO, EAU, and the European Society for Therapeutic Radiology and Oncology (ESTRO) to create a joint committee to develop uniform recommendations for the follow-up of both non-invasive and invasive TCC patients. This would improve the early diagnosis of local recurrence, metastasis, and SPMs, and ultimately achieve better treatment outcomes.

Our study has several limitations that should be acknowledged. Firstly, the relatively small sample size may limit the applicability of the findings to larger or more diverse populations. Secondly, as a retrospective study, there is an inherent risk of selection bias and incomplete data, which may affect the accuracy and interpretation of results. Thirdly, we lacked comprehensive data on important confounding factors such as smoking status, comorbidities, genetic background, blood tests, and specific treatment regimens, all of which could influence metastatic patterns. The lack of these data limits the ability to evaluate potential associations or underlying causes of SPMs. We recommend that future prospective studies incorporate these variables to allow for more comprehensive risk assessment. Additionally, variability in follow-up imaging may have impacted the detection rates of MTS. While both computed tomography (CT) and positron emission tomography and computed tomography (PET-CT) were used during follow-up, it is important to note that PET-CT is generally more sensitive than conventional CT in detecting metastatic disease, especially in LNs. This difference in imaging sensitivity could have influenced the observed rates of LN MTS in our study, potentially leading to earlier or more accurate detection in patients who underwent PET-CT. We acknowledge the limitation regarding the lack of detailed breakdown between PET-CT and CT imaging modalities. In this study, imaging was performed based on clinical availability and patient suitability; therefore, some patients underwent PET-CT while others had CT alone. Unfortunately, precise distribution data were not consistently recorded across all cases. We recognize that imaging modality could influence detection sensitivity, and we recommend that future studies standardize imaging protocols and report modality-specific data to allow for a clearer understanding of its impact on diagnostic outcomes. Another limitation of our study was the inability to perform subgroup analysis based on age. Lastly, the study was conducted in a single-center setting, which may not reflect broader geographic or population-based trends.

## 5. Conclusions

This study highlights distinct patterns of metastatic spread in TCC patients, differing notably from previously reported trends in SPMs. These differences may reflect underlying geographic, genetic, lifestyle, and healthcare-related factors. Understanding these variations is essential for tailoring patient management strategies and improving outcomes through more personalized surveillance and treatment approaches.

## Figures and Tables

**Table 1:** Patient characteristics.

Eligible Patients	90
Median age	70 (range 22–87)
Primary treatments:	
Radical cystectomy	58 patients (64%)
Median age	66 (range 43–86)
Partial cystectomy *	9 patients (10%)
Median age	64 (range 22–73)
Radiotherapy	23 patients (26%)
Median age	74 (range 22–87)

* Five patients were additionally treated with XRT.

**Table 2:** The distribution of metastases.

Metastases Development	66 Patients
Sites of MTS:	
Pelvic LNs	34 patients (52%)
Bone	18 patients (27%)
Liver	8 patients (12%)
Lung	6 patients (9%)
Synchronous LNs + Liver *	4 patients (6%)
Average time from diagnosis to MTS	14.3 months

* In addition to the primary study groups, we included patients who had synchronous lymph node and liver metastases.

**Table 3:** The distribution of MTS with respect to treatment.

Radical Cystectomy	Number of Patients
Lymph node	17
Liver	7
Bone	6
Lung	3
**Radiotherapy (XRT)**	
Lymph node	17
Bone	12
Lung	3
Liver	1

**Table 4:** The average time for metastasis development.

Site	Time (Months)	Hazard Ratio (95% Confidence Interval)	*p* Value
Bone	6.8	3.25 (2.1–5.0)	<0.001
Lymph node	14.8	1.82 (1.1–3.0)	0.015
Lung	16	1.70 (0.95–3.1)	0.071
Liver	59.7	Reference	—

**Table 5:** The risk of metastasis based on treatment.

Metastatic Site	Radical Cystectomy (%)	Radiotherapy (%)	*p* Value
Lymph node	51.5%	51.5%	1.0
Bone	18.2%	36.4%	0.10
Liver	21.2%	3.0%	0.05
Lung	9.1%	9.1%	1.0

**Table 6:** Follow-up of patients with non–MIBC (EAU guidelines) [37].

Recommendation	GR
The follow-up is based on regular cystoscopy.	A
Patients with low-risk tumors should undergo cystoscopy at 3 months. If negative, subsequent cystoscopy is advised 9 months later, and then yearly for 5 years.	C
Patients with high-risk tumors should undergo cystoscopy and urinary cytology at 3 mo. If negative, subsequent cystoscopy and cytology should be repeated every 3 months for a period of 2 years, and every 6 months thereafter until 5 years, and then yearly.	C
Patients with intermediate-risk tumors should have an in-between follow-up scheme using cystoscopy and cytology, which is adapted according to personal and subjective factors.	C
Regular (yearly) upper tract imaging (CT-IVU or IVU) is recommended for high-risk tumors.	C
Endoscopy under anesthesia and bladder biopsies should be performed when office cystoscopy shows suspicious findings or if urinary cytology is positive.	B
During follow-up in patients with positive cytology and no visible tumor in the bladder, R-biopsies or biopsies with PDD (if equipment is available), and investigation of extravesical locations (CT urography, prostatic urethra biopsy) are recommended.	B

Abbreviations: CT, computed tomography; GR, grade of recommendation; IVU, intravenous urography; PDD, photodynamic diagnosis; R-biopsies, random biopsies.

## Data Availability

Data are contained within the article or are available from the authors upon reasonable request.
